# Simultaneous Coronary Artery Bypass Grafting and Anterior Rectum Resection: The First Clinical Case in Kazakhstan

**DOI:** 10.15388/Amed.2024.31.2.8

**Published:** 2024-12-04

**Authors:** Anuar Abdikarimov, Serik Aitaliyev, Vladimir Dikolayev

**Affiliations:** 1 JCS “National Scientific Medical Center”, Astana, Kazakhstan; 2 Department of Cardiac, Thoracic and Vascular Surgery, Hospital of Lithuanian University of Health Sciences Kaunas Clinics, Lithuanian University of Health Sciences, Kaunas, Lithuania; Faculty of Medicine and Health Care, Al-Farabi Kazakh National University, Almaty, Kazakhstan; 3 Multifunctional hospital No 2, Astana, Kazakhstan

**Keywords:** colorectal cancer, ischemic heart disease, simultaneous treatment, Raktažodžiai: storosios žarnos vėžys, išeminė širdies liga, gydymas vienu metu

## Abstract

In this case report, we describe the experience of a patient who was initially admitted for rectal cancer treatment. However, during the preoperative evaluation, severe stenosis of the coronary arteries was unexpectedly detected, presenting the medical team with a complex decision-making process.

## Abstract (it)

Aprašome paciento, kuris iš pradžių buvo paguldytas gydyti tiesiosios žarnos vėžio, patirtį. Prieš-operacinio įvertinimo metu netikėtai buvo aptikta sunki vainikinių arterijų stenozė, todėl gydytojų komandai teko priimti sudėtingą sprendimą.

## Introduction

The coexistence of cardiovascular diseases and oncologic conditions is increasingly prevalent worldwide, and this rise can be attributed to advancements in diagnostic methodologies and the aging population [1]. Patients initially presenting with one clinical state could be diagnosed with an additional unexpected condition during preoperative assessment. This dual diagnosis presents clinicians with a complex dilemma: what treatment should be taken in advance? Can one procedure be delayed, or is it feasible to address both conditions simultaneously? Resolving these questions requires a tailored approach for each patient, as there is a lack of relevant guidelines due to the wide spectrum of possible diagnoses and comorbidities. The resolution depends on disease specifics and the patient’s overall health [8].

Previous research has shed light on the potential benefits of simultaneous treatment, such as conducting both surgeries under a single anesthesia, providing an opportunity to address both conditions concurrently, and alleviating the distress associated with prolonged illness. However, simultaneous procedures pose inherent risks, including an increased potential for complications and adverse outcomes due to the substantial physiological stress induced by concurrent surgeries [5]. As a result, deciding on the most appropriate management of these dual conditions remains an unanswered question, characterized by a lack of well-defined criteria for determining the optimal treatment course.

## Case report

In this report, we present the case of a 71-year-old female patient admitted to the surgery department of the National Scientific Medical Center, Astana, Kazakhstan, with primary complaints of bloody loose stools, general weakness, and abdominal bloating. The patient signed the informed consent form for collecting and storing information related to her medical history as well as for the simultaneous surgery (coronary artery bypass graft surgery plus laparoscopic anterior resection of the rectum).

**Figure 1:**
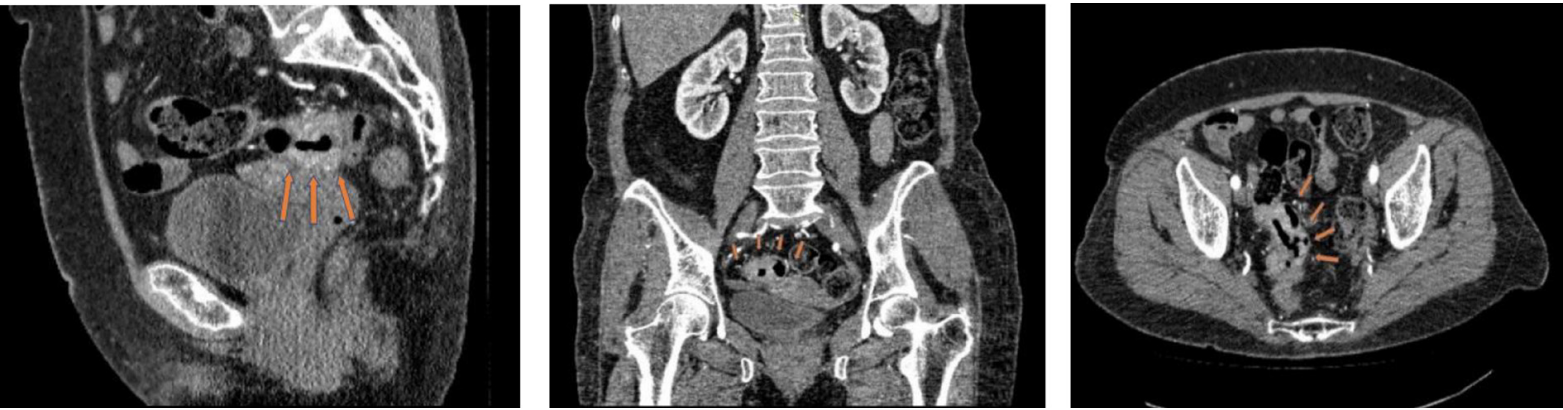
Pelvic CT colon with tumor, the arrows indicate tumor (a) sagittal projection, (b) anterior projection, (c) axial projection.

The patient had been experiencing symptoms for two months, which had worsened over time. After visiting an outpatient clinic and consulting with an internal physician, the patient underwent a colonoscopy, during which a mass in the rectosigmoid part of the colon was discovered. The subsequent biopsy confirmed the presence of moderately differentiated adenocarcinoma, leading to the patient being referred to the oncology unit for further treatment. A laboratory analysis revealed anemia, which was likely due to bleeding from the tumor site (Figure 1). Further blood tests showed results within normal ranges.

The electrocardiogram (ECG) showed left ventricular hypertrophy but no conduction problems. The abdominal contrast-enhanced computer tomography (CT) scan revealed a critical stenosis of the celiac trunk (95%), requiring a consultation with the interventional radiologist. However, there was no clinically significant sign revealed. Therefore, no intervention was done. During the physical examination, the patient appeared well with no abnormal findings. However, a rectal examination revealed fresh blood on the glove.

**Figure 2:**
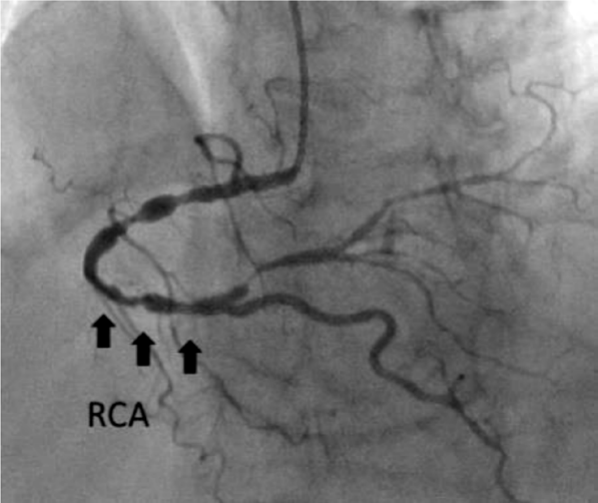
Coronarography. RCA: stenosis 90–95%.

**Figure 3:**
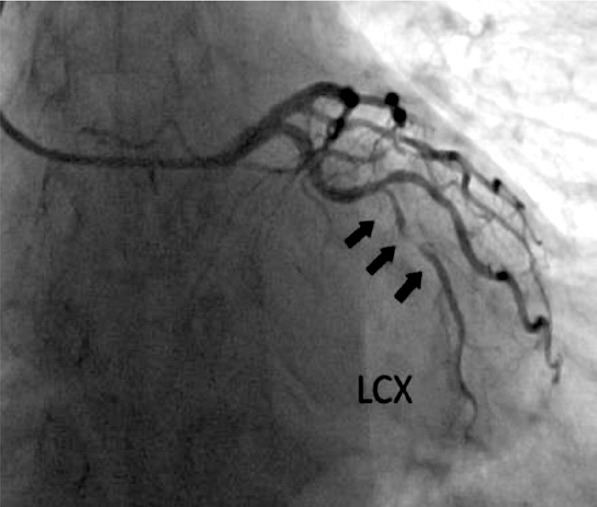
Coronarography. LCX: distal left circumflex coronary artery stenosis 95%.

**Figure 4:**
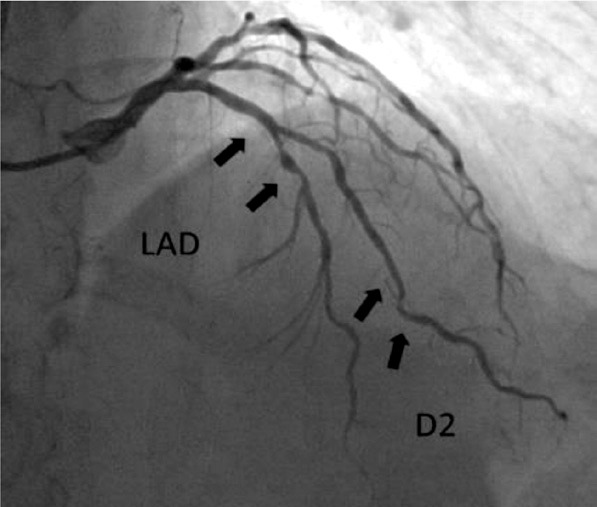
Coronarography. LAD: left anterior descending artery stenosis 95%. D2: the second diagonal artery.

While obtaining the patient’s medical history, it was discovered that the patient had been experiencing chest pain for several years. The patient had a history of arterial hypertension for several decades and had received periodic consultations with a cardiologist for medication adjustments. However, living in a rural area, the patient had never undergone an echocardiogram or other diagnostic procedures. A cardiologist consulted before surgery and a coronarography of the heart was performed, which revealed severe obstructive coronary artery disease: distal left circumflex artery (LCx) – 95%; distal right coronary artery (RCA) – 90–95%; proximal posterior left ventricular branch (PLb) – 95% stenosis (Figures 2, 3, and 4).

After a discussion with the multidisciplinary team, considering the localized tumor in the rectosigmoid part of the colon with bleeding, the patient was advised to undergo simultaneous surgery to address both the heart disease and rectal cancer in a single procedure. However, despite the doctors’ recommendations, the patient declined this treatment and was discharged.

After a two-month period, the patient returned to the clinic and provided the consent to undergo simultaneous surgery. The patient’s condition remained stable, with no significant changes observed in laboratory and instrumental analyses.

The simultaneous surgery commenced with a team of cardiac surgeons performing coronary artery bypass grafting (CABG) using a cardiopulmonary bypass. The procedure included the mammary coronary bypass of the left anterior descending artery (LAD), autovenous bypass of the obtuse marginal artery, and posterior interventricular artery. The duration of the cardiopulmonary bypass was 48 minutes, with a cross-clamp time of 20 minutes. Intraoperative bleeding was 200 ml.

**Figure 5:**
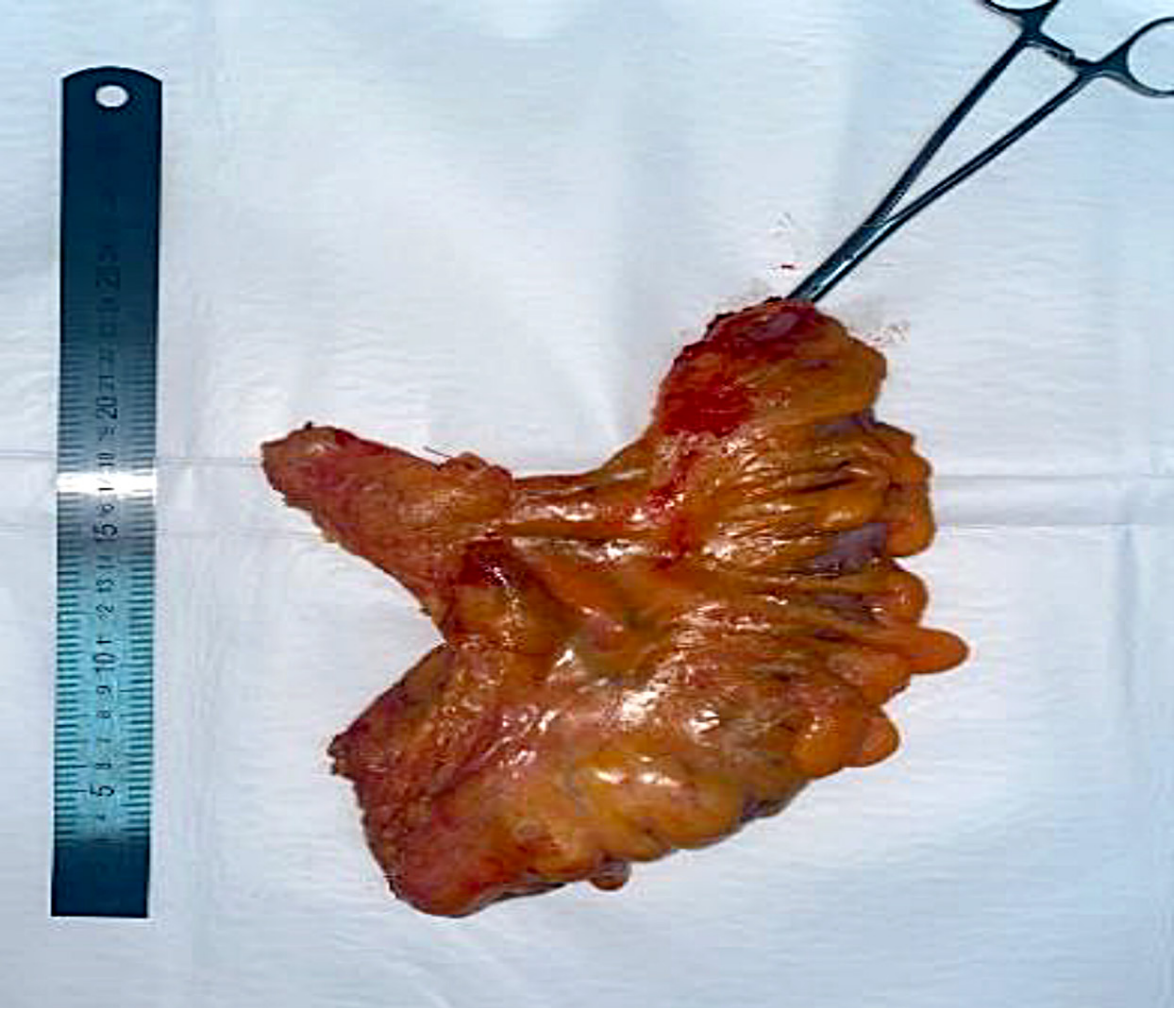
Macropreparation of the resected colon with tumor.

Following the cardiac step, a team of general surgeons performed a laparoscopic anterior resection of the rectum with subsequent anastomosis. The tumor measured 5x6x7 cm in size, and the specimen was resected within the limits established by oncologic protocols (>Figure 5). The total duration of the combined surgery was 310 minutes. After completion, the patient was transferred to the intensive care unit (ICU) without requiring inotropic support and was successfully extubated 7 hours after the surgery. The patient received close monitoring in the ICU for one day before being transferred to the surgical ward.

The histologic evaluation of the tumor revealed moderate differentiated adenocarcinoma of the colon (G2) with a spread to the entire wall thickness, with focal shallow ingrowth into adipose tissue (pT3N0). Distal and proximal edge fragments of the colon wall were with morphological signs of chronic colitis, there were no sign of malignant tumor growth. Furthermore, tumor growth in nine lymph nodes was not detected. Rehabilitation procedures were initiated, and the patient was discharged in a satisfactory condition on the 8th day after surgery. The final diagnosis was ischemic triple vessel heart disease and rectosigmoid cancer T3N0M0 St IIa, Duke B.

## Discussion

Patients with colorectal cancer often require surgery as part of their treatment, but there may be other comorbidities that can complicate the surgical approach. Similarly, patients with severe coronary artery disease may require surgery, but it can be challenging if they have other health conditions. Managing patients with both coronary artery disease and colon cancer poses a dilemma for physicians as these two conditions require contradictory treatment approaches. Both surgeries carry high risks of hemorrhagic, cardiac, infectious, pulmonary, renal and gastrointestinal complications [1,2]. Patients with dual diseases, consecutive surgeries can carry an extremely high risk of mortality. In such situations, simultaneous surgery can help to mitigate many risks. However, while simultaneous surgeries can be enticing in certain cases, it is crucial to carefully consider each patient’s overall health status and potential complications associated with factors such as age, sex, and other health conditions.

It was found that out of 4,079 patients referred for cardiac surgery, 103 of them (2.5%) had a history of cancer [3]. Currently, there are no established guidelines for the treatment of these patients. A previous literature search revealed various cases describing different treatment approaches. For instance, one case involved a patient with both colon cancer and a heart tumor who underwent an anterior resection for the sigmoid tumor. Two weeks after the initial surgery, the patient successfully underwent heart surgery with positive outcome >[4].

In another study, a series of nine patients underwent simultaneous surgery for their cardiac and cancer-related conditions. The study reported no significant difference in outcomes between the two treatment approaches. However, it is important to note that this result cannot be considered statistically significant due to the very low number of patients included in the study [5]>.

In the decision-making process involving such patients, it is crucial to approach each case individually. For instance, in the case mentioned, a two-stage surgery was not viable due to the patient’s existing bleeding from the rectal tumor. The use of heparin during CABG would likely have resulted in a fatal outcome. Moreover, the patient’s critical coronary artery stenosis posed a high risk of mortality during colon surgery.

Another potential treatment option would have been to perform percutaneous transluminal coronary angioplasty (PTCA) prior to colon surgery. However, in this particular case, it was not a feasible choice as the patient was already experiencing bleeding from the tumor site. Additionally, a previous study suggested that coronary stenting increases the risk of ischemic cardiac events during subsequent surgeries, even with the recommended six-week interval [6]. Another study proposed that the optimal time between percutaneous coronary intervention (PCI) and surgical intervention is three months, which minimizes the risk of in-stent thrombosis [7].

While some sources may recommend CABG as the initial treatment approach, it is important to consider the potential drawbacks. CABG as the primary step can significantly prolong the recovery period, during which the tumor may have the opportunity to spread. As a result, numerous studies now advocate for simultaneous surgeries as the preferred method of treating patients with concomitant diseases.

Due to advancements in surgical techniques and the development of surgical instrumentation, simultaneous surgeries are increasingly being performed worldwide. In the case of heart and lung diseases, simultaneous surgeries have become well-established and are almost considered a standard treatment approach [8].

It is evident that there is still limited knowledge regarding the optimal care for patients with concomitant diseases. One potential concern among surgeons is the addition of clean-contaminated surgery to cardiac surgery, which may increase the risk of infections and sepsis. However, no literature data were found regarding this specific issue, and in the case of our patient, no infection was present. We can, therefore, conclude that by adhering to clear recommendations regarding sterilization techniques, surgical approaches, antibiotic prophylaxis, and wound care, the development of infectious processes can be adequately prevented.

In simultaneous surgeries involving both heart and colorectal cancer, there is an ongoing debate regarding whether to use off-pump or on-pump surgery due to concerns about possible tumor spread. However, other studies have shown that using cardiopulmonary bypass (CPB) does not carry an increased risk of the disease spreading [9].

Furthermore, other sources support the notion that both off-pump and on-pump coronary artery bypass grafting yield superior outcomes compared to percutaneous coronary intervention [10]. Additionally, a study has indicated that off-pump heart surgery can help minimize the risk of potential tumor spreading [11].

At present, the survival rate following simultaneous surgeries is heavily influenced by the nature and stage of the tumor [12]. The relatively poorer prognosis observed in patients with lung and gastrointestinal diseases, as well as the comparatively better prognosis in patients with kidney tumors, suggests that patient survival is greatly influenced by the underlying biology of the malignancy.

## Conclusion

This report presents the clinical case of an elderly patient with concomitant heart and colon disease who underwent simultaneous surgery, including CABG and laparoscopic anterior resection. The postoperative period went well with no complications. The patient was discharged with recommendations and referred for further rehabilitation.

Our case report presents a possible treatment scenario for a patient with both coronary artery disease and colon cancer. The chosen treatment method is consistent with many prior studies and was the best option for our patient. There is still a significant amount of research required on this topic since there are still no clear recommendations worldwide. Further investigations should be performed, especially controlled trials, to analyze postoperative functional and surgical results.

## Data Availability

Data will be available under request to the corresponding author.
